# Geographic mapping and spatiotemporal patterns of tuberculosis in Libya within ten years' period (2015 to 2024)

**DOI:** 10.3389/fepid.2025.1571065

**Published:** 2025-09-11

**Authors:** Mohamed Ali Daw, Abdallah H. El-Bouzedi, Saleh Ali Abumahara, Abdurrahman Khalifa Najjar, Nouri R. Ben Ashur, Alaa Grebi, Amnnh Mohammed Dhu, Ali Fathi Alkarghali, Shahid Husayn Mohammed, Raja Khalid Miftah, Najmuldin abdulbasit abdulsamad, Mohammed Saad Elbasha, Asawer Seifennaser Doukali, Nosieba Taher Elmhidwi, Esra Othman Albouzaidi, Said Emhamed Wareg, Mohamed Omar Ahmed

**Affiliations:** ^1^ Department of Medical Microbiology & Immunology, Faculty of Medicine, University of Tripoli, Tripoli, Libya; ^2^ Clinical Microbiology & Epidemiology, Acting Physician of Internal Medicine, Scientific Coordinator of Libyan Society of Hospital Infection, Tripoli, Libya; ^3^ Department of Statistics, Faculty of Science, Tripoli University, Tripoli, Libya; ^4^ Faculty of Medicine, Department of Surgery, Tripoli, Libya; ^5^ Faculty of Medicine, Department of Gynecology, Tripoli Medical Centre, Tripoli, Libya; ^6^ Faculty of Medicine, University of Tripoli, Tripoli, Libya; ^7^ Department of Biology, University of Nalout, Nalout, Libya; ^8^ Department of Microbiology & Parasitology, Faculty of Veterinary Medicine, University of Tripoli, Tripoli, Libya

**Keywords:** tuberculosis (TB), case notification rate, geographic patterns, temporal trends, Libya

## Abstract

**Introduction:**

Tuberculosis(TB) is still a serious problem with a remarkable global impacts particularly within developing countries such as Libya. According to World Health Organization (WHO) global report, the country is considered a moderate TB burden with incidence of 40 per 100,000 in 2011. Geographic epidemiology has been considered an important tool in preventing TB in large countries. In this study, we intended to identify the geographic and spatiotemporal patterns of the TB incidence rate in Libya between 2015 and 2024.

**Methods:**

A cross-sectional retrospective analytical study was conducted within ten years on the data reported through the National TB surveillance system. The data on all TB cases reported from 2015 to 2024 by municipality and region was abstracted. Choropleth maps were drawn showing the TB case notification rates (CNR) per 100,000. Local Moran's I was performed to identify the spatial variations of the disease and temporal and Spatiotemporal analyses were employed in all instances.

**Results:**

During the entire study period, 26,478 TB cases were reported from all 22 municipalities in Libya with an annual rate of 40.29/100,000 (95% CI: (40.229 ± 9.01). The highest incidence was reported in 2015 and the lowest one in 2024. Males were significantly reported more than females among notified TB cases, (*P* < 0.001). The highest CNR was reported in the Eastern region followed by Western and Southern regions. The geospatial distribution of reported cases of TB varied greatly within the provinces and during the study period. There was evident variability throughout the country and over time. High-rate and low-rate clusters were predominantly distributed in the periods. High clusters were concentrated northeast and northwest, though low-level clusters were mainly located in the middle and the southern region of the country.

**Conclusion:**

The results of this study provided clear insights into the geographic and spatiotemporal mapping of TB in Libya. There was an overall decreasing trend in TB CNR from 2015 to 2024 parallel with high-risk and low-risk areas. This information should allow the decision-making personnel to implement proper policies to combat TB at national and regional levels.

## 1 Introduction

Tuberculosis (TB), is still one of the most serious health threats, particularly in developing countries. It is considered to be the world's leading cause of death from a single infectious agent and caused almost twice as many deaths as HIV/AIDS. Over 10 million people continue to fall ill with TB every year and the number has been increasing since 2021 (1).

Different studies have shown that the incidence of certain infectious diseases particularly TB has spatial aggregation. Geographic epidemiology is a new branch of epidemiology, which is a discipline to describes and analyzes the geographical distribution of diseases (2). It does apply geographic visualization technology and highlights the spatial distribution patterns. Geographic epidemiology can analyze the geographical analysis of diseases in the study area, as to obtain the scope and changes of disease aggregation, which should be a key role in health decision-making. However, up to now, not many studies have been reported on TB in terms of economic and medical security (3, 4).

Spatiotemporal studies from different countries demonstrated that TB has highly complex dynamics and is spatially heterogeneous at provincial, national, and international levels during certain periods. In China, such studies identified seasonal patterns and spatial-temporal clusters of TB cases at the county level. The most likely clustering time was spring, and the most likely clustering areas were the southeast and northeast regions (5). Another spatiotemporal study on TB carried out in Pakistan identified high-risk clusters across the country during 2015 to 2019, especially in the northern and western parts of the study area (6).

In central Africa, Masabarakiza et al. carried geographic study in Burundi during the 2009–2017 period and reported that the eastern parts of Burundi had experienced a relatively low incidence rate of TB compared to other parts of the country (7). Another retrospective study from Western Kenya, analyzing the spatial distribution of 23,374 TB cases from 2012 to 2015, noted that the TB incidence varied from 638.0 to 121.4 persons per 100,000 at the small-area level (8). Similar studies conducted in Ethiopia from 2007 to 2016 revealed strong variation of TB from 70.4 to 155.3 persons per 100,000 population (9).

However, such studies are rarely carried out in Northern African countries. A short Spatiotemporal distribution study for only three years (2011–2014) was carried out in Morocco indicating that, TB is not randomly distributed in space and only two distinctive spatial regimes that affect TB spatial clustering were identified (10). Libya is the second largest country in Africa with the longest coast in the Mediterranean facing Europe bordered by six countries, with a population of only 6.7 million. The country is considered a major transit and destination country for international migration (11). While the majority of migrants in Libya report that they originally intended to stay and work in Libya, the country has become an important departure point for migrants to Europe (11, 12). It has been speculated that immigration from areas of high TB incidence is thought to have fueled the resurgence of TB in Libya (13, 14). A study carried out on the prevalence of TB among asylum seekers in Italy, concluded that the highest prevalence of 535 per 100,000 population was reported from asylum seekers in Bologna. This was explained by the different nature of migration flows and extended stay in detention and overcrowding for several months in Libya before reaching Italian coasts, which might increase the risk of TB transmission and the risk of progression from infection to disease (15). Therefore, studying the geographic epidemiology of TB in Libya become an important priority not only for the country itself but also for Mediterranean and European countries.

Libya is one of the rare countries that started documentation and tracing of TB in 1950, Fossati et al. reported on the mortality of pulmonary TB in Libyan children in Cirenaica (Libya) from 1959 through 1964 (16–18). TB remains a public health challenge in Libya, with the country classified as a moderate TB burden country by the WHO. While the national TB program has reported a decline in some areas, this is largely attributed to increased detection among migrants. There is also a significant TB burden within the Libyan population, with an increase in cases from 40 per 100,000 population in 2013 to 59 per 100,000 in 2015, and remaining at 59 per 100,000 in 2021.

In recent years, geographic epidemiology and spatial-temporal analysis have been widely used by Daw and his research team to describe the distribution characteristics and transmission patterns of infectious diseases in Libya including Hepatitis viruses, HIV/AIDS, and COVID-19 (19–21). In this study, we conducted a geographic and spatiotemporal analysis of TB in Libya within ten years from 2015 to 2024 to trace the spread and deposition of TB within this vast geographic area. Further to evaluate the effectiveness of prevention programs and highlight strategies needed to combat the spread of TB.

## 2 Materials and methods

### 2.1 Study setting

**Figure 1:**
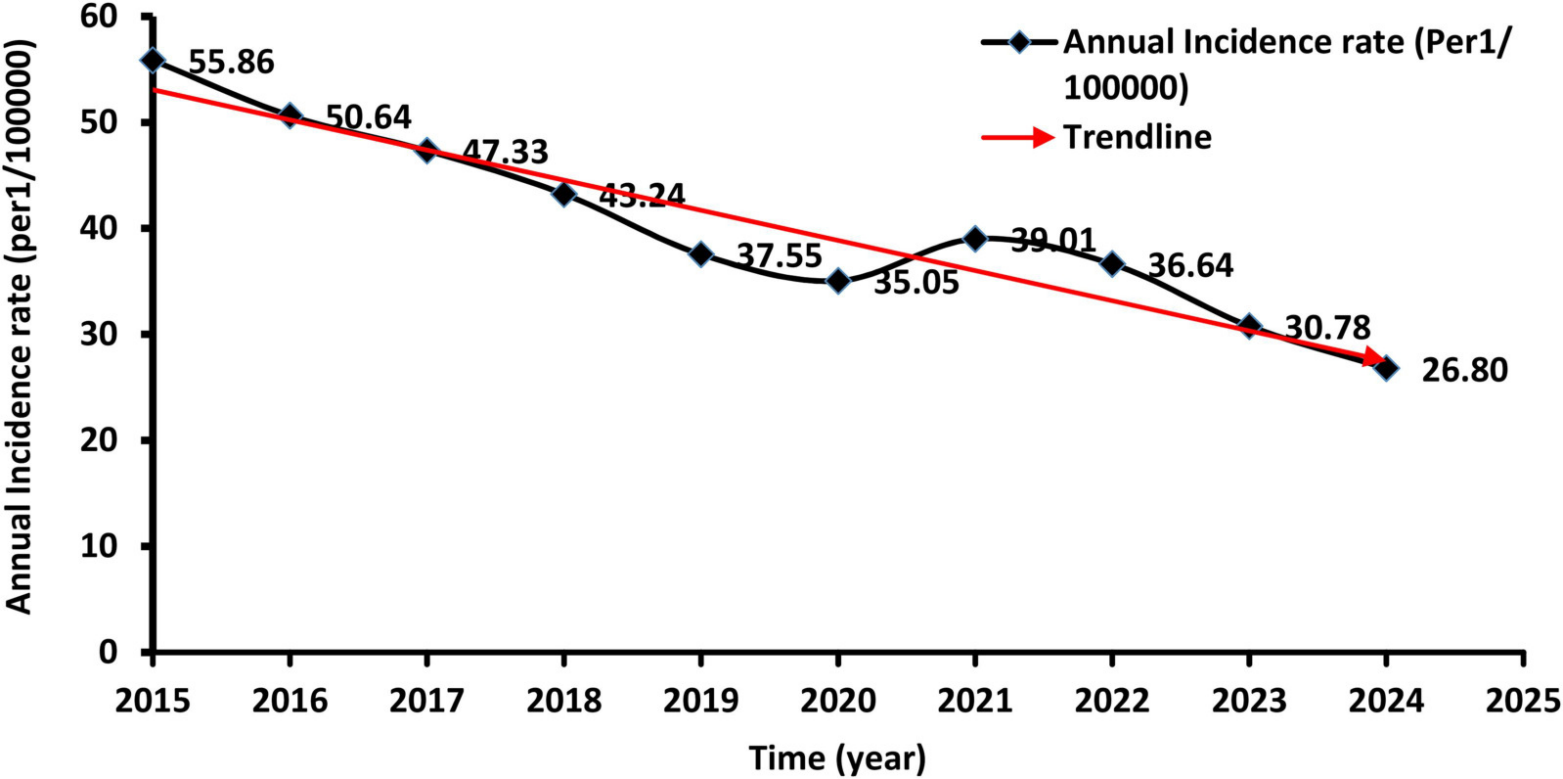
Annual incidence rate of tuberculosis reported in Libya from 2015 to 2024.

In 2024 Libya has an estimated population of 7,381,023. The country is divided into three administrative regions (Eastern, Southern, and Western) with 22 municipalities as shown in Figure 1. Its territory spans 1,759,540 Km^2^–4 per Km^2^ (679,362 sq. miles) making it the second largest country in Africa. Libya's coastline is 1,770 km, the longest coast in the Mediterranean basin. 77.3% of the population is urban (5,703,871 people in 2024). The study period was from January 01, 2015, to 31 December 2024, and covered a total of 26,478 patients at the provincial level across all of Libya. For each year under study, the population density by municipality was calculated (22). The study included all TB notifications in Libya during the study period and all country municipalities were involved.

### 2.2 Data collection

The data was captured from the Libyan National TB program registry. This covers all 22 provinces (municipalities) of Libya and collects data on all TB case notifications reported by hospitals during the study period. Using (WHO) definitions and reporting framework for TB (23). Laboratory diagnosis of all suspected individuals was detected by microscopy of Ziehl-Neelsen-stained sputum smears. All non-pulmonary TB patients were recognized by pathologic methods used regularly in Libyan hospitals. Data on both new cases and the incidence of TB by municipality were collected. The demographic information as well as medical information were collected from each patient (24). Each case was identified separately and the names and identities of the patients were augmented during data extraction to protect patients' privacy.

### 2.3 Geographic and spatial analysis

Geographic analysis was carried out by Spatial scan statistics using SaTScan™ (25) to detect statistically significant clusters for high-risk TB in the municipalities where participants were recruited. SaTScan imposes circles of different sizes on the geographic area and computes a likelihood ratio statistic based on the number of observed and expected cases within and outside the circle and compared with the likelihood under the null hypothesis. The analysis was undertaken at each municipality and the prevalence of TB was calculated per 100,000 population for each one. Furthermore, Relative risk (RR) was calculated in each cluster to evaluate the risk of TB within the identified boundaries of each municipality. A *p*-value. Global Moran's I values calculated by ArcGIS v.10 software (ESRI Inc., Redlands, CA, USA) were used to identify spatial autocorrelation and detect the spatial distribution pattern of TB in all twenty-two municipalities in Libya (26). Global Moran's I is more sensitive to departures from the null hypothesis, which assumes that TB is randomly distributed in the area under study. The range of Moran's I value range is between [−1, 1]. A positive Moran's I value indicates that a positive correlation exists, and the larger the value, the more obvious the tendency to cluster is, while a negative Moran' I value indicates that a negative correlation exists, showing a discrete distribution. There is no spatial clustering when the value is zero, meaning that the data are randomly distributed. Both the Z-score and *P*-value are used to evaluate the significance of Moran's I (27, 28).

## 3 Results

**Figure 2:**
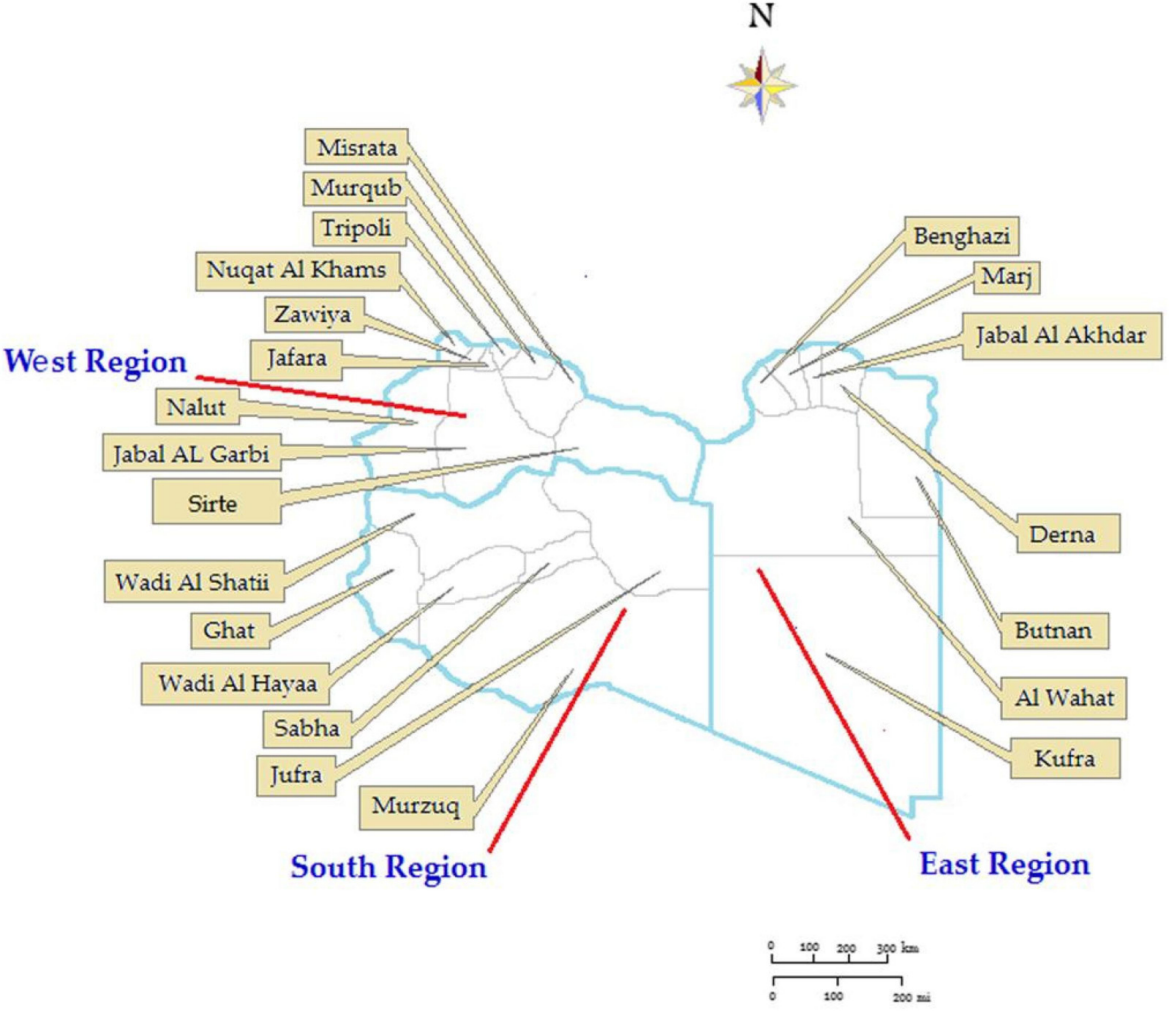
The geographical locations and boundaries of the Libyan regions and municipalities.

A total of 26,478 TB cases were notified in Libya over the entire study period which lasted for ten years (2015 to 2024). Table 1 shows the number and the annual reported incidence rate. With an average annual reference rate of 40.29/100,000 [95% CI: (40.229 ± 9.01)/100,000]. (trend *χ*^2^ = 20.67, *P* < 0.014). Figure 2 illustrates the TB incidence per 100,000 people during the study period. The incidence showed a downward trend year by year. The highest incidence was reported in 2015 and the lowest one in 2024. There was a 3% annual variation of TB CNR reported from 2015 (55.86/100,000 population) to 2024 (26.80/100,000 population) (*p*-value for trend *p* < 0.014). There was overall, case notification rates decreased during this study period (Figure 2).

**Table 1:** Reported incidence of tuberculosis in Libya from 2015 to 2024.

Years	Cumulative number of cases	Annual incidence rate (Per1/ 1,00,000)	Mean	CI:95%	*P*-Value
2015	3,458	55.86			
2016	3,181	50.64			
2017	3,018	47.33			
2018	2,801	43.24			
2019	2,467	37.55	40.29	40.229 ± 9.01	0.014
2020	2,332	35.05			
2021	2,627	39.01			
2022	2,496	36.64			
2023	2,120	30.78			
2024	1,978	26.80			

In 2019 and 2020 there was a decrease in TB CNR to 37.55 and 35.05/100,000 respectively then it rose to 39.01/100,000 in 2021. The demographic characteristics of TB-reported cases in Libya from 2015 to 2024 are shown in Table 2. The annual average notification rate among males was significantly higher than that of females (*P* < 0.001). The highest notification rate was reported among people aged over 60 years old.

**Table 2:** Demographic characteristic of notified cases of tuberculosis in Libya −2015 to 2024.

Characteristic	Study period									
	2015	2016	2017	2018	2019	2020	2021	2022	2023	2024
Gender										
Male	1,954	2,112	2,029	1,985	1,859	1,407	1,818	1,807	1,311	1,215
Female	1,504	1,069	989	816	608	925	809	698	809	763
Age(year)										
≤10	79	73	61	47	64	43	75	57	49	41
11–20	231	240	197	109	97	87	101	98	89	73
21–30	491	242	207	237	188	119	183	112	107	95
31–40	607	513	548	411	373	305	387	294	265	197
41–50	711	741	627	597	446	392	513	511	495	329
51–60	520	550	477	479	471	571	537	603	508	520
>60	819	822	901	921	828	815	831	821	611	723

**Figure 3:**
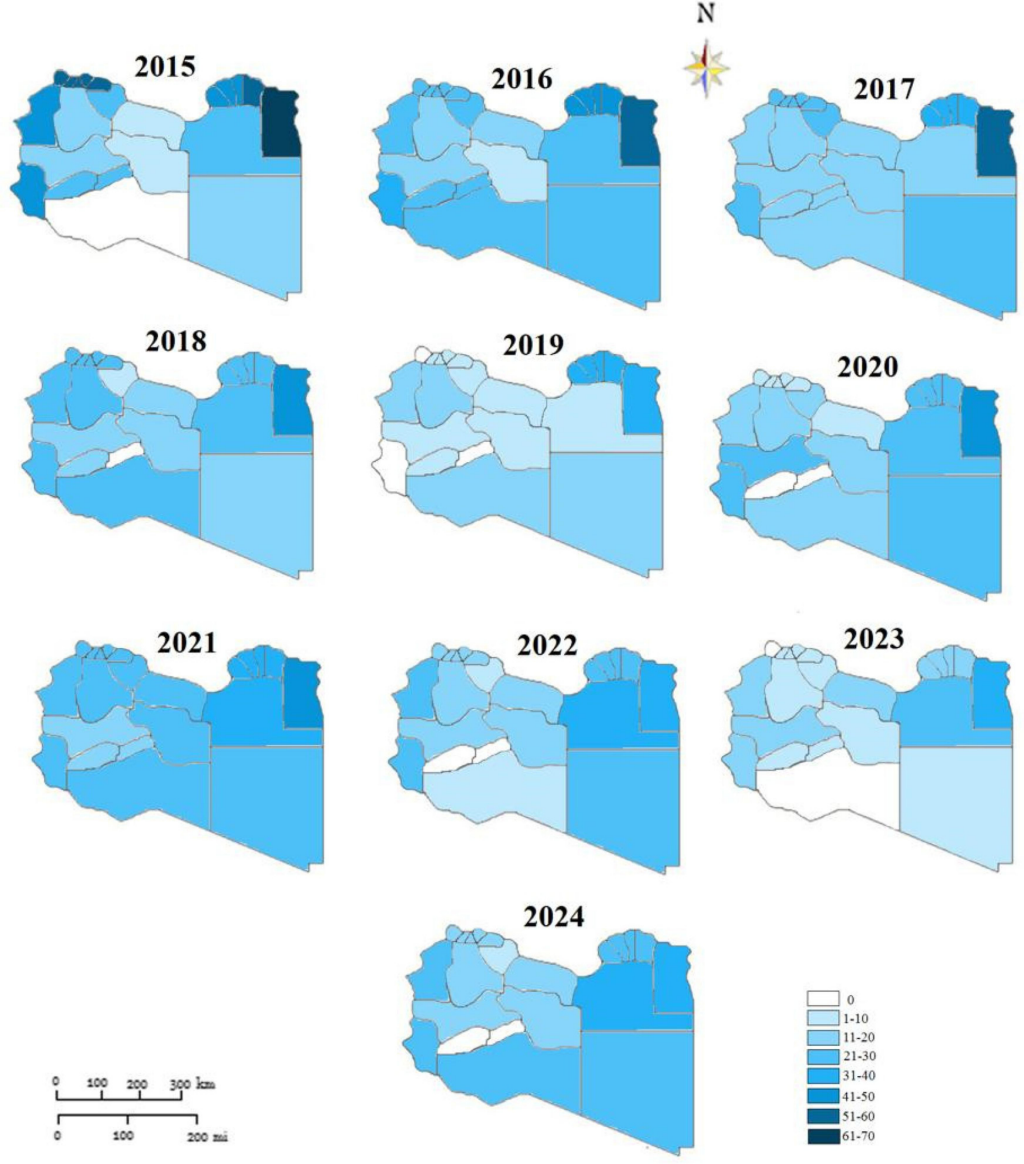
Geographic mapping of tuberculosis case notification in Libyan districts-2015 to 2014 (rates per 1:100,000).

The Libyan regions showed variable CNR across the ten-year study period as shown in Figure 3. The highest CNR was reported in the Eastern region particularly, Albtnan, Derna, Aljabel Alkader, and Benghazi, followed by the Western region particularly Zawai, Nugat Alkhams (Zawara), and Tripoli followed by Nalut and Western Mountain (Jabal Al-Karbi). However, the Southern region has the lowest CNR, particularly Jufra and Murzuq.

The results indicate that the high rate of total TB was predominantly distributed in the period between 2015 and 2017. In terms of areas with low rates, TB clustering was distributed between 2021 and 2024 but was predominantly identified in 2021 and 2022.

The spatial distribution of CNR was approximatively stable from 2015 to 2017 in Butana, Derna, and Jabal Akhdar in the Eastern region. While the variation was noticeable in the Western region. Although low TB incidence rates were located throughout the southern part of the country during the study period. It was observed that TB incidence was decreasing over all the country but not in the same way in all areas.

The global autocorrelation Moran's index of the incidence rate of TB in all municipalities of Libya from 2015 to 2024 is shown in Table 3. The largest Moran's index was reported in 2017(Moran's I = 0.5134), while the global Moran's index was the smallest in 2019((Moran's I = 0.2541). The *P* value was <0.05, indicating that the annual TB distribution of all provinces and cities in Libya has an obvious positive spatial correlation. At the same time, the overall Moran's I index shows an upward trend, indicating that the concentration of development levels in various provinces and cities is increasing year by year.

**Table 3:** Spatial autocorrelation analysis of incidence rate of tuberculosis in Libya from 2015 to 2024.

Year	Moran's index	Z-value	*P*-value
2015	0.3,152	2.14	0.0323
2016	0.4723	2.88	0.0132
2017	0.5134	3.12	0.0089
2018	0.4.232	2.75	0.01.53
2019	0.2541	1.95	0.05.32
2020	0.3420	2.11	0.0371
2021	0.4123	2.65	0.0221
2022	0.5002	3.05	0.01.05
2023	0.2875	2.11	0.045.1
2024	0.3453	2.34	0.03.22

**Figure 4:**
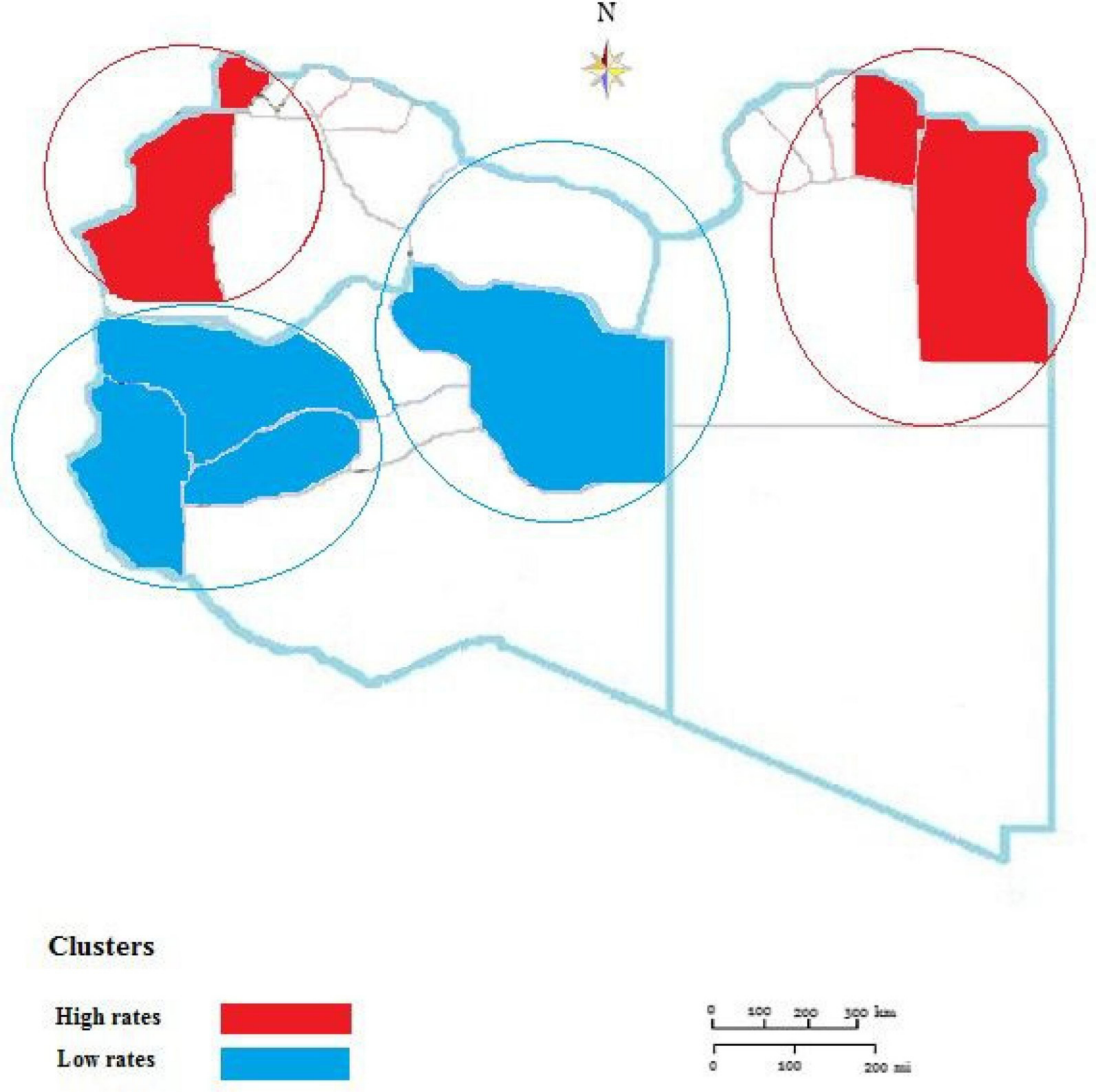
Spatiotemporal clustering of tuberculosis in Libya from 2015 to 2014.

During the study period from 2015 to 2024, geospatial statistical analysis results showed high and low-risk clusters scanned as shown in Figure 4. The high-risk clusters are concentrated northeast including 4 municipalities (Butnan, Derna, Jabal Al Akhdar, Marj) and 3 municipalities in the northwest including [Zauara (Nuqat Al Khams), Nalut, Jabal- Al Garbi]. Low-level clusters were mainly located in Meddle and the southern region of the country including 8 municipalities(Jafara, Zawiya, Sirte, Ghat, Wadi Al Hayaa, Al-Wahat, Kufra.

## 4 Discussion

The application of geographic and spatiotemporal analysis for microbial diseases has greatly enhanced our understanding of the epidemiology of important diseases such as TB, Viral hepatitis, and HIV/AIDS both on global and local scales (29–31). These studies were widely carried out in China and America to frame and contain the spread of TB. However, they were rarely used in Africa, particularly in the Northern region (32–34). Libya is one of the largest countries in Africa and less populated with a population density of 4/Km^2^. The geographic entity of this country makes it difficult to control the spread of contagious diseases, particularly TB. This study provides some great insights into the geographic and spatiotemporal epidemiological status of TB in Libya over the last ten years. The results identified a decreasing trend of TB during the period 2015 to 2024. During this period Libya reported 26,478 cases of TB, with an average annual incidence rate of 39.78/100,000. The annual reported incidence rate showed a downward trend year by year, with an average annual decline of 4.12%. The western region had the most obvious change and the Southern region provinces in Libya were at a low level during the ten years. These results are in concordance with other studies carried out in Iran, and China as there was a clear variation in the geographic distribution of TB within the regions of these large countries (35, 36).

Libya is surrounded by six countries which considered to be endemic by TB. This study showed the highest TB rates were reported in Albtanan, Derna, and Aljabel Alkader in the Eastern region closer to Egypt. Followed by Western Mountain and Zowara in the Western region neighboring Tunisia. This is in agreement with other studies carried out in other African country. Kapwata et al. reported that in South Africa clusters with high-risk ratios for TB were detected in regions where TB data were collected with the most likely clusters being located around border areas (37, 38). This could be attributed to socioeconomic, immigrant settlements, and poor health services (39–41). Hence then further studies are needed to substantiate this assertion. However, efforts should be implemented at regional and national levels by applying screening to immigrants, refugees, and workers (42, 43).

During this study period (2015 to 2014), a total of four clusters were reported including 15 municipalities. The clustering areas are mainly concentrated in the northeast and northwest and the display of this agglomeration area was closely related to the borders of the neighboring countries. This is in agreement with studies reported in other African countries including South Africa and Kenya (8, 44). However, further studies are needed on the other socio and economic factors associated with the geographic deposition and concentration of TB (45, 46).

### 4.1 Strengths and limitations

To the best of our knowledge, this is the first study carried out in Northern African countries particularly in Libya to analyze the geographic epidemiology of TB incidence rate in the country as a whole. The study was carried out not only at the regional level but even at the finer geography scale of each municipality. However, despite our ability to determine the geographic variability and intensity of TB in this vast country. It should be acknowledged that this study has several limitations. First, our data analyses do not contain all TB cases identified in Libya in the time frame of interest and it was not possible to analyze additional variables such as underlying clinical conditions. Second, the study is retrospective, and there may be certain selection bias that affects the results as it makes it difficult to more specifically explain the observed trends. Third, we were not able to consider the impact of the novel coronavirus epidemic, and Libyan internal armed conflict particularly as they evolved parallel during the study period. 2019–2020 (47, 48).

## 5 Conclusion

This study indicated clearly that determining geographic mapping and spatial distribution of TB incidence rate could be a useful tool for guiding the policymakers regarding the high-risk provinces and municipalities to implement needed national and regional preventive and combat policies according to geo-variability and spatial distribution of such infectious disease. However, further studies are needed to assess the sociodemographic factors, particularly within the municipalities with a high-density rate at the Eastern and western regions (49, 50).

### 5.1 Recommendations

The geographic mapping applied in Libya in this study should be applied at a large scale to include other Northern and sub-Saharan African countries that are known to be endemic, particularly cross borders regions. Furthermore, these countries are known to be the main hub of immigrants both as a source of immigration and transient cross-area toward Europe. Therefore, controlling TB should priority by screening passengers and immigrants (51–54).

## Data Availability

The datasets presented in this article are not readily available. Requests to access the datasets should be directed to mohamedadw@gmail.com.
